# Extensive Renal Arteriovenous Malformations Treated by Transcatheter Arterial Embolization

**DOI:** 10.1155/2017/2376034

**Published:** 2017-01-31

**Authors:** Tadashi Tabei, Hironao Tajirika, Jun Yoshigi, Kazuki Kobayashi

**Affiliations:** ^1^Department of Urology, Yokosuka Kyosai Hospital, Kanagawa, Japan; ^2^Department of Radiology, Yokosuka Kyosai Hospital, Kanagawa, Japan

## Abstract

An 84-year-old woman was referred to our department due to gross hematuria. Enhanced computed tomography revealed early enhancement of the right renal vein and multiple tortuous vessels around the right renal hilus, part of which had invaded into the renal parenchyma and renal calix. We diagnosed her with arteriovenous malformations (AVMs) and performed transcatheter arterial embolization (TAE). Angiography showed extensive and complex AVMs located in the central and peripheral areas of her kidney. After TAE, the hematuria resolved and she became hemodynamically stable.

## 1. Introduction

Congenital renal arteriovenous malformations (AVMs) are abnormal communications between the intrarenal artery and vein via a cluster of multiple, enlarged, tortuous arteriovenous communications, called a vascular nidus [1]. It is a rare cause of gross hematuria, with a prevalence of less than 1% among the general population [2]. In recent years, transcatheter arterial embolization (TAE) has replaced surgical treatment [3] for AVMs. Although TAE is a minimally invasive treatment option, it sometimes requires an expert technique, especially for complicated cases. We present a case of extensive AVMs treated by TAE.

## 2. Case Presentation

An 84-year-old woman was referred to our department due to gross hematuria. Her abdomen was soft and flat, and costovertebral angle tenderness was not present. No vascular bruit was audible on her abdomen. She had a history of asthma, skin cancer, and hypertension, for which she took 4 mg of candesartan daily. Although she had previously experienced gross hematuria around age 40 years, the details of that episode were not known. Cystoscopy showed hematuria flowing off the right ureteral orifice. Plain computed tomography (CT) showed an intrarenal high-density lesion. Contrast-enhanced CT and intravenous pyelography were not undertaken because of her history of asthma. Retrograde pyelography revealed no suspicious formation in the right ureter or pelvis. In addition, right pelvic urine cytology was class I. The patient was diagnosed with an idiopathic renal hemorrhage.

**Figure 1:**
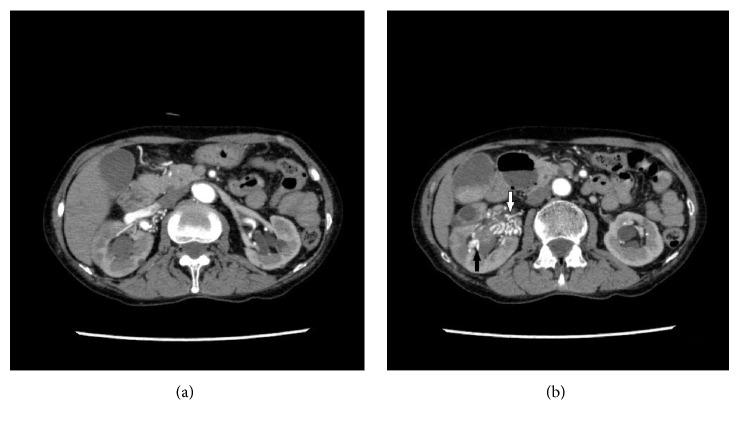
(a) The right renal vein was enhanced in the early phase. (b) There are multiple tortuous vessels around the right renal hilus (white arrow), part of which invaded into the renal parenchyma and renal calix (black arrow).

**Figure 2:**
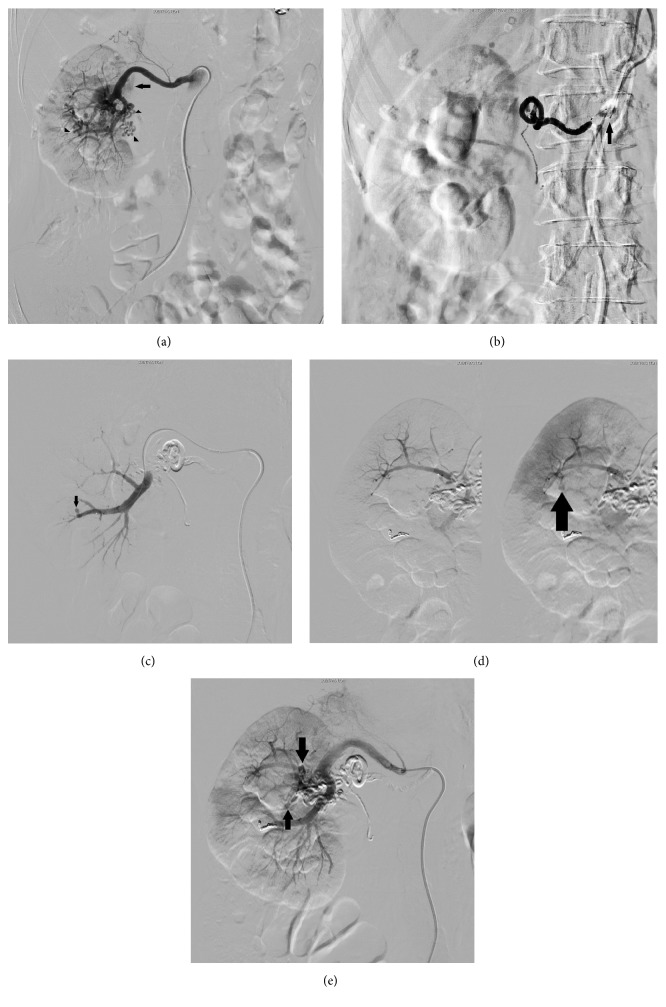
(a) Angiographic image obtained prior to embolization. Abnormal tortuous vessels (nidus) arise from the right renal artery (arrow heads). Early enhancement of inferior vena cava can be seen (arrow). (b) Embolization of abnormal vessels is performed from the ovarian artery using 0.8 cc of n-butyl-2-cyanoacylate (NBCA) lipiodol under arterial flow control by occlusion balloon (arrow). (c) Abnormal vessels from the renal lower segmental branch are embolized using a gelatin sponge and metallic coils. Extravasation to collecting system can be seen (arrow). (d) Extravasation to collecting system from the renal upper segmental branch (arrow). (e) Postembolization angiography. Minor abnormal vessels remain (arrow).

Since her general condition was stable and laboratory data showed slight anemia (hemoglobin [Hgb], 9.6 g/dL), conservative treatment was chosen. However, the gross hematuria persisted for a week, and laboratory test results revealed exacerbation of the anemia (Hgb 5.8 g/dL). Following the results of conservative treatment, a blood transfusion was initiated, and enhanced CT was performed after consulting with a respiratory disease expert. CT revealed the early enhancement of the right renal vein (Figure 1(a)) and multiple tortuous vessels around the right renal hilus, part of which had invaded into the renal parenchyma and renal calix (Figure 1(b)). There was no apparent neoplasia on her right kidney. We diagnosed the patient with AVM, and emergent angiography was performed. There were abnormal vessels arising from the right renal artery (Figure 2(a)). Embolization of the abnormal vessels from the ovarian artery was performed using 0.8 cc of n-butyl-2-cyanoacylate (NBCA) lipiodol (NBCA : lipiodol = 1 : 4) under arterial flow control by occlusion balloon (Figure 2(b)). Extravasation via abnormal vessels from the renal lower segmental branch was resolved using a gelatin sponge and metallic coils (Figure 2(c)). Although there were no apparent abnormal vessels, extravasation from the renal upper segmental branch was found, which was embolized with a gelatin sponge (Figure 2(d)). Minor abnormal vessels remained (Figure 2(e)). However, those vessels were difficult to select with a catheter and were not involved in the extravasation. The gross hematuria was resolved, and complete hemostasis was achieved.

Therefore, total occlusion was resigned to prevent a wide range of renal ischemia secondary to the nonspecific embolization. The estimated glomerular filtration rate on the day following TAE was 51.3 mL/min/1.73 m^2^ (it had been 52.8 mL/min/1.73 m^2^ before TAE). She was discharged 8 days after TAE.

## 3. Discussion

We presented a case of AVM showing gross hematuria. Although AVM is a rare cause of gross hematuria, the majority of patients with AVM (72% of cases) experience gross hematuria as a primary symptom [4]. Renal AVMs are divided to three types, including the cirsoid, angiomatous, and aneurysmal [1]. The cirsoid AVM is the most common type, characterized by knotted, tortuous vessels called a “nidus.” In contrast, aneurysmal AVMs are composed of a single feeding artery and draining vein. Aneurysmal AVMs are difficult to differentiate from chronic acquired arteriovenous fistulas, which are usually caused by renal trauma [4]. In the present case, angiography showed multiple feeding arteries and draining veins, which suggested the cirsoid type of AVM. Additionally, the patient had no history of trauma or renal surgery. Therefore, we diagnosed her with congenital AVM.

AVM is more often observed in women than in men, and it is found more often in the right kidney than in the left [5, 6].

In the present case, the patient was female, and the AVM was observed in the right kidney, as in previous reports. Her age at onset was older than in those reports. Generally, abnormal vessels invading under the urothelial mucosa leads to gross hematuria in the second to fourth decades of life [5]. However, she reported experiencing gross hematuria when she was around 40 years old. Assuming that the hematuria was caused by the AVM, her history is in line with the reported peak age of onset.

In recent years, surgical treatment for AVM has rarely been performed, having been replaced by TAE, which allows the occlusion of the vascular lesion [3]. Since TAE is a more selective treatment than simple nephrectomy, it can preserve renal function of the affected side. In this case, the procedure was more challenging because of the extensive and complex lesions. There are several embolization agents, including the gelatin sponge, coils, absolute alcohol, and NBCA.

In recent years, NBCA has become the most popular agent, for several reasons. First, NBCA can produce permanent obliteration. Moreover, its conjugation time can be controlled by alerting the lipiodol concentration. However, in complicated cases, such as this one, combination use of those agents is sometimes more effective than using NBCA alone to prevent a wide range of infarction and to decrease the procedure time [7].

Total occlusion of abnormal vessels is recommended in order to prevent recurrence. However, Takebayashi et al. [3] reported partial occlusion may be sufficient to stop hematuria even in large and complex lesions because it is effective in reducing renal venous pressure and occlusion of ruptured veins into the collecting system. Actually, partial occlusion achieved complete hemostasis in the present case. Although impaired renal function following multiple embolizations was a concern, the patient's renal function was not changed severely. TAE is an effective treatment for AVM, even in cases with complex lesions, as in this case.
